# Olfactory Interference during Inhibitory Backward Pairing in Honey Bees

**DOI:** 10.1371/journal.pone.0003513

**Published:** 2008-10-23

**Authors:** Matthieu Dacher, Brian H. Smith

**Affiliations:** School of Life Sciences, Arizona State University, Tempe, Arizona, United States of America; Freie Universitaet Berlin, Germany

## Abstract

**Background:**

Restrained worker honey bees are a valuable model for studying the behavioral and neural bases of olfactory plasticity. The proboscis extension response (PER; the proboscis is the mouthpart of honey bees) is released in response to sucrose stimulation. If sucrose stimulation is preceded one or a few times by an odor (forward pairing), the bee will form a memory for this association, and subsequent presentations of the odor alone are sufficient to elicit the PER. However, backward pairing between the two stimuli (sucrose, then odor) has not been studied to any great extent in bees, although the vertebrate literature indicates that it elicits a form of inhibitory plasticity.

**Methodology/Principal Findings:**

If hungry bees are fed with sucrose, they will release a long lasting PER; however, this PER can be interrupted if an odor is presented 15 seconds (but not 7 or 30 seconds) after the sucrose (backward pairing). We refer to this previously unreported process as olfactory interference. Bees receiving this 15 second backward pairing show reduced performance after a subsequent single forward pairing (excitatory conditioning) trial. Analysis of the results supported a relationship between olfactory interference and a form of backward pairing-induced inhibitory learning/memory. Injecting the drug cimetidine into the deutocerebrum impaired olfactory interference.

**Conclusions/Significance:**

Olfactory interference depends on the associative link between odor and PER, rather than between odor and sucrose. Furthermore, pairing an odor with sucrose can lead either to association of this odor to PER or to the inhibition of PER by this odor. Olfactory interference may provide insight into processes that gate how excitatory and inhibitory memories for odor-PER associations are formed.

## Introduction

Insects have proven to be invaluable for studying not only the basic forms of learning but also for understanding how higher-order cognitive processes might be supported by their smaller, more accessible nervous systems [1]–[3]. Use of insects such as fruit flies and honey bees has clearly provided insight into the cellular and molecular events that underlie sophisticated behavioral plasticity in higher vertebrates [4], [5]. Likewise, well-conceived mechanistic studies of insect behavior can reveal components of the more complex cognitive phenomena found in mammals [6]–[11].

In particular, there is a rich history of using the honey bee for studying both non-associative and associative learning processes [1]–[3], [12]–[17] as well as cognitive processes such as choice behavior [18], [19], non-elemental learning [20], [21], the use of conceptual rules [9]–[11], spatial orientation [22], [23], visual categorization [24], delayed alternation [25], lateralization [26] and inter-individual communication about location of food via the ‘dance language’ [23], [27]. Non-associative and associative learning have been extensively studied in the laboratory with restrained bees using a well-defined behavioral response called the proboscis extension response (PER, the proboscis is the main mouthpart of the bee). PER is an appetitive response triggered when sucrose solution -the unconditioned stimulus (US)- is applied to the antennae and/or the proboscis. The animal then extends its proboscis to consume the sucrose solution. If an odor –the conditioned stimulus (CS)- precedes and slightly overlaps sucrose presentation (forward pairing), the bee will form an excitatory association between this odor and the sucrose (and/or the PER) by way of classical (Pavlovian) conditioning [28]. On the other hand, presentation of an odor 15 s after sucrose delivery (backward pairing) will produce inhibitory learning about this odor, i.e. bees will display poorer performance during subsequent training [29].

The PER olfactory conditioning protocol allows for the precise control of stimulation parameters in behavioral studies as well as for simultaneous linkage to neurophysiological and imaging analyses of brain activity [30]–[33]. Furthermore PER conditioning allows for pharmacological and molecular manipulation of identified modulatory pathways involved in processing stimuli, learning and reinforcement [34]–[48]. Recent studies have begun to extend PER conditioning to cognitive processes such as choice behavior [18], [19] and non-elemental learning [20], [21] using restrained bees (rather than free-flying bees) in the controlled conditions of a laboratory. These studies provide the opportunity for associating more complex kinds of learning with neurophysiological measurements and molecular manipulations.

Backward pairing of sucrose and odor has received relatively less attention [29]. Here we report on the observation of a new PER related phenomenon, which we call olfactory interference. During the olfactory interference protocol, a hungry bee consumes a small droplet of sucrose solution, which elicits PER that continues for several seconds after the sucrose has been consumed. After the end of the feeding, the proportion of bees displaying the ongoing PER decays smoothly. This ongoing PER can be abruptly interrupted by presentation of an odor stimulus (backward pairing), but only within a specific time frame. Olfactory interference elicits inhibitory conditioning as with other backward pairing stimuli [29]. We argue that studying olfactory interference will provide insight into an important process that gates how excitatory and inhibitory memories for odor-PER associations are formed in the brain. It is well-established that inhibitory neurotransmitters (such as histamine) are involved in olfactory processing in well defined areas of the honey bee brain [33], [37], [48], [49]. We show that olfactory interference can be disrupted by blockade of cimetidine-sensitive pathways specifically in the deuterocerebrum.

## Results

### Olfactory interference

**Figure 1:**
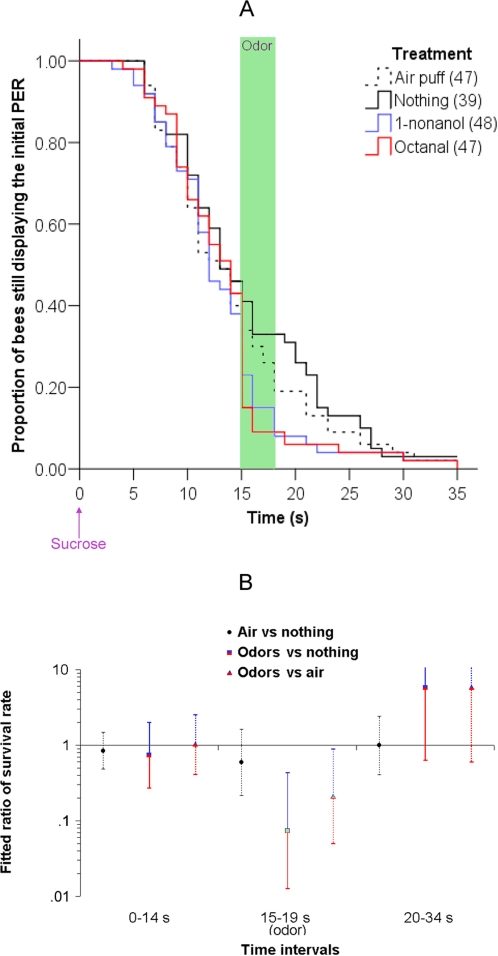
Olfactory interference: time course of the PER (A) and fitted ratio of survival rate (B). (A) Proportion of bees which are still displaying the initial PER as a function of the time after the onset of sucrose feeding (which started at 0 s). There is one curve for each of the four experimental groups: animals presented with an air puff, with no treatment (control group), with 1-nonanol or with octanal. The air puff or the odors were delivered 15 s after the onset of the sucrose feeding and lasted 4 s; this is indicated by the green area on the plot. Values in parenthesis are the number of animals used in each group. In subsequent figures, the odors are presented pooled because they never differed significantly. (B) Same data as in A, displayed in three sections: before odor onset (0–14 s), during odor presentation to one second after (15–19 s) and after the odor (20–34 s). For each of these time periods, Cox regression provided three ratios of the PER survival rates. Within each group, the PER survival rate is the proportion of bees that did not stop displaying a PER during a given time period. The Cox regression estimates fitted ratios of these PER survival rates. The ratios that are represented are (survival in the air-treated group)/(survival in the control group), (survival in the odor-treated groups)/(survival in the control group), and (survival in the odor-treated groups)/(survival in the air-treated group). A 95% confidence interval is determined for each ratio, illustrated by the error bars. The ratios odor/air and odor/control are significantly lower than 1 during the 15–19 s time period (the odor presentation period), which means that the odor-treated groups have a significantly lower “survival” rate than the air-treated or the control group during the odor presentation; in other words, they retract their proboscis more often. Note that the scale is logarithmic. This is because Cox regression uses an exponential equation that produces very large (and asymmetric) error bars. Furthermore, the error bars increase in size when the number of animals still displaying a PER decreases at later time points, because the sample size is decreasing and the estimation of the ratio loses precision. Hence, the error bars are larger during the last time period (19–34 s) because few bees are still displaying a PER.

In this first experiment, sucrose was presented to the bee to release PER. A 4 s odor pulse (either 1-nonanol or octanal) was presented 15 s after the onset of feeding. We used a delay of 15 s because previous work indicated that backward inhibitory conditioning occurs at this interval [29]. Two control groups of bees received either no stimulation (nothing group) or just air (i.e. the air was blown across an unscented filter paper inside the syringe). The behavior of the bees was videotaped to measure the duration of the initial PER (see materials and methods and supplementary Figure S1 for details). The results are presented Figure 1.

In the “nothing” and “air-only” groups, the probability of continuing with the initial PER declined with time. This is is the normal response to sucrose stimulation. The bees initially responded to sucrose stimulation with a PER lasting 3–7 s, which corresponds to the time needed to consume the sucrose. After that, the proportion of bees extending their proboscis declined at a fairly constant rate through 30–35 s, by which time most bees had stopped the PER. There were no statistically significant differences between the air-only group and the nothing group (Wald test: before the odor, 0–14 s: χ^2^ = 0.335, p = 0.553; during the odor, 15–19 s: χ^2^ = 1.019, p = 0.313; after the odor, 20–34 s: χ^2^ = 0.0002, p = 0.989). Therefore, the air alone did not have any effect on the probability of continuing the PER, although the performance in this group is slightly lower than in the untreated group.

Olfactory interference is clearly apparent in groups that received odor stimulation. In both groups that received an odor there was a sharp decline in PER at the onset of the odor relative to the rate of decline in the two control groups. This decline was evident relative to the “nothing” control during odor presentation (Wald test during odor, 15–19 s: χ^2^ = 8.297, p = 0.004) but not before or after (before the odor, 0–14 s: χ^2^ = 0.360, p = 0.548; after the odor, 20–34 s: χ^2^ = 2.417, p = 0.120). Similarly, the odor group was significantly different from the air-only group when the odor was presented, but not before or after odor presentation (Wald test; before the odor, 0–14 s: χ^2^ = 0.004, p = 0.952; during the odor, 15–19 s: χ^2^ = 4.460, p = 0.035; after the odor, 20–34 s: χ^2^ = 2.333, p = 0.127), indicating that the olfactory component of stimulation (rather than the mechanosensory component from air) is more salient for retraction of the proboscis. This is consistent with previous reports that the mechanical effect of air that carries the odorant is much less salient for the bee than the odor itself [15], [50]; we also observed that bees placed in a constant air flow still display olfactory interference (data not shown). In summary, an odor presented 15 s after sucrose feeding can terminate the ongoing PER: this is the olfactory interference effect (see supplementary Movie S1). The observation of olfactory interference has been replicated in supplementary Figure S2, which demonstrates its reliability. We also presented sucrose to the antennae alone (rather than on both antennae and proboscis) without feeding the bees. In that case, the bees quickly retracted the proboscis, which prevents the detection of olfactory interference (supplementary Figure S3).

### Time dependency of olfactory interference

**Figure 2:**
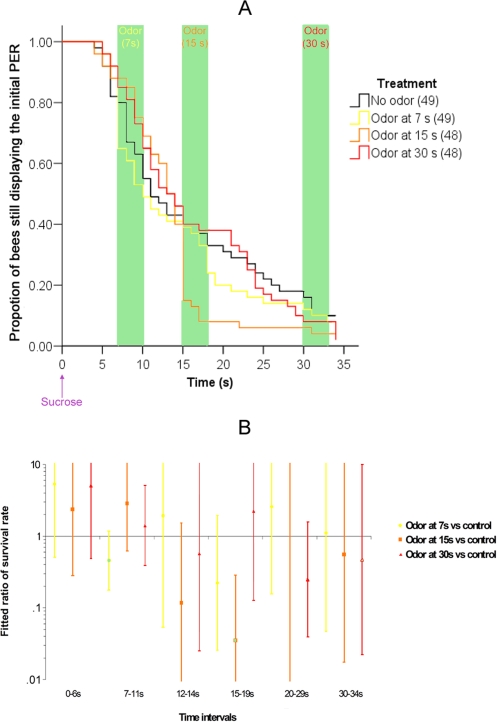
Olfactory interference tested at 7, 15 and 30 s: time course of the PER (A) and fitted ratio of survival rate (B). This figure is similar to Figure 1, except that the four different experimental groups were: odor presented at 7 s, odor presented at 15 s, odor presented at 30 s, and no treatment. Therefore, each group was stimulated once with the odor (at 7 s, 15 s or 30 s), or not at all (control group). The odor was either 1-nonanol or octanal, and the results for both odors are presented pooled because they were not significantly different. In part B, the time-periods correspond to before any treatment (0–6 s), during to one second after the odor presentation in the 7 s group (7–11 s), the between period (12–14 s), during to one second after the odor presentation in the 15 s group (15–19 s), the between period (20–29 s) and during to one second after the odor presentation in the 30 s group (30–34 s). All other details are as in Figure 1.

We evaluated the time frame for olfactory interference by stimulating different groups of bees with odor (1-nonanol or octanal,) 7, 15 or 30 s after onset of feeding (Figure 2). Consistent with the previous observation (compare to Figure 1), when the odor was presented 15 s after the onset of sucrose feeding olfactory interference was observed as a sharp decline in PER associated with odor stimulation. This group was significantly different from the control (no odor) group (Wald test; no odor group vs. odor at 15 s group, during the 15–19 s period: χ^2^ = 9.838, p = 0.002). On the other hand, olfactory interference was not found when the odor was presented 7 s or 30 s after the sucrose (Wald test; no odor group vs. odor at 7 s group, during the 7–11 s period: χ^2^ = 2.627, p = 0.105; no odor group vs. odor at 30 s group, during the 30–34 s period: χ^2^ = 0.236, p = 0.627). Therefore, olfactory interference occurs specifically at or around 15 s after the onset of feeding. The decrease observed when the odor was presented at 7 s was also observed in the control group, but the difference between the two groups was not significant. This decrease can be explained by the spontaneous retraction of the proboscis occurring in some bees after the end of feeding (i.e. not all the bees release a long-lasting PER).

Among bees that had stopped extending their proboscis before the odor onset, we rarely observed proboscis extension when odor was presented. That would have been an indication of “sensitization” documented in other studies of PER in flies and bees for odors presented 30 s after sucrose stimulation [15], [41], [47], [51]–[54]. Sensitization should lead to a transient rise in proportion of bees responding during or shortly after odor presentation at 30 s (or at the two other delays). Although we observed a small increase in the proportion of bees responding, it was not significantly different from the control group (10.3% of the bees resumed the PER at 30 s in the 30 s group vs. 2.5% in the control group; Fisher's exact test, p = 0.360).

### Olfactory interference, sucrose sensitivity and sensitization

This experiment had two objectives. First, we wanted to confirm the absence of sensitization-induced odor responses by presenting the odor 30 s after the sucrose. Second, we wanted to assess whether well established genetic differences among bees in sucrose sensitivity would affect olfactory interference and the sensitization-induced odor response. Therefore, we used both nectar foragers and pollen foragers, as pollen foragers are more sensitive to sucrose than nectar foragers [55]; the previous experiments used only nectar foragers.

**Figure 3:**
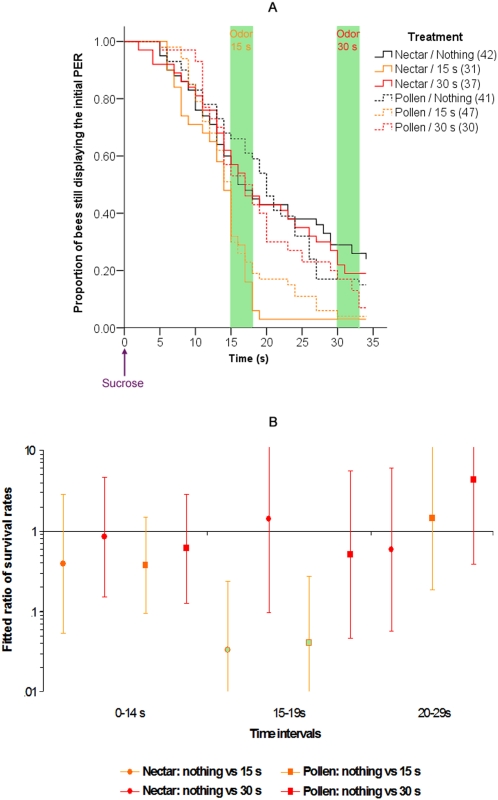
Olfactory interference with nectar and pollen foragers (A, B). This experiment is similar to the one shown in Figure 2, except that both pollen and nectar foragers were used, and that the odor was presented at 15 s, 30 s, or not at all (control group). A, B: details are as in previous figures. In part B, some points are missing because the Cox regression did not converge to a solution for them due to the very low number of bees still displaying a PER after 30 s in the 15 s groups.

Bees in each foraging group (nectar or pollen) were either presented with 1-nonanol or octanal 15 s or 30 s after sucrose presentation, or they were left untreated (Figure 3). The results were the same for both pollen and nectar groups: animals that were presented with either odor abruptly retracted their proboscis when exposed to odor 15 s after the onset of feeding (Wald test; nectar foragers, no odor group vs. odor at 15 s group, during the 15–19 s period: χ^2^ = 11.463, p = 0.0007; pollen foragers, no odor group vs. odor at 15 s group, during the 15–19 s period: χ^2^ = 10.894, p = 0.00097). In addition, animals presented with an odor at 30 s did not resume the PER more often than the control group (nectar groups: 3.6% in the control group vs. 13% in the 30 s group, Fisher's exact test, p = 0.329; pollen groups: 4.3% in the control group vs. 5.6% in the 30 s group, Fisher's exact test, p = 1), so neither nectar nor pollen foragers displayed a sensitization-induced odor response in our experimental conditions. This led us to conclude that olfactory interference is equivalent in nectar and pollen foragers, which suggests that olfactory interference is not affected by sucrose sensitivity.

There was only a small difference between the pollen and nectar untreated groups: while they were not different in the 15–19 s time period (Wald test: χ^2^ = 0.198, p = 0.656), pollen foragers tended to retract their proboscis more often after this time (Wald test: χ^2^ = 5.374, p = 0.020). This indicates that pollen foragers tend to retract their proboscis slightly earlier than nectar foragers.

### Olfactory interference is associated with backward inhibitory learning

**Figure 4:**
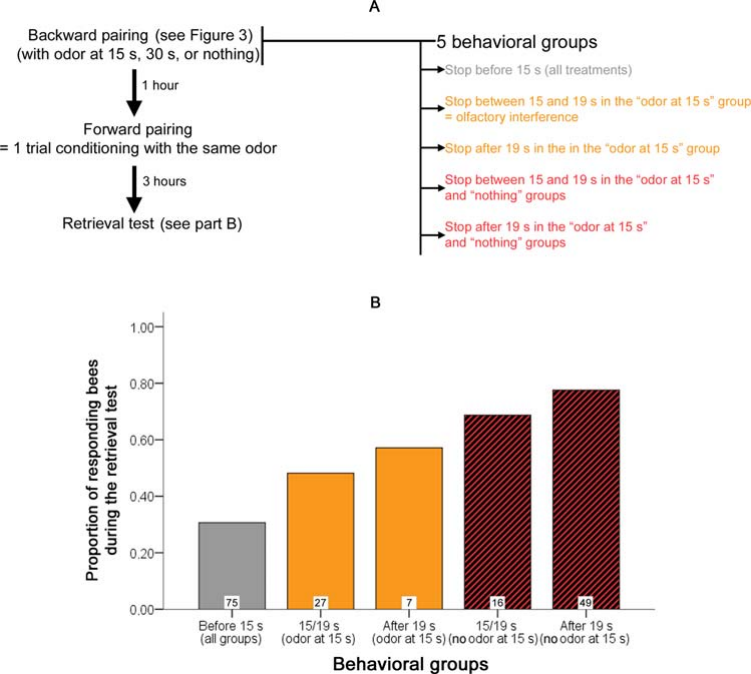
Effect of olfactory interference on a subsequent learning. (A) Details of the protocol used. One hour after the backward pairing, the bees were trained with a single forward pairing, followed by a retrieval test 3 hours after the training. The odor used was either 1-nonanol or octanal. For the 15 s and the 30 s groups, the same odor was used for backward and forward pairing. The animals that were trained in this forward pairing are those of Figure 3 (see this figure to their performance during olfactory interference). Although the same animals were used as in A, the sample size is lower because some bees died or did not respond to the sucrose after backward pairing (the proportion of bees discarded for these reasons was the same across the six groups: 5 degrees of freedom χ^2^ = 1.8624, p = 0.868, and the olfactory interference effect was also significant for the remaining bees). After conducting the whole experiment, animals were assigned to one of five behavioral groups according to the time at which they retracted their proboscis during the backward pairing (see details in figure). These five groups are used for the analysis in part B. B: Performance of the bees during the retrieval test, as a function of their behavioral group. The boxes at the base of each bar indicate the sample size. An alternative representation is provided in supplementary Figure S4; this figure also provides a justification for the attribution of the groups in part A.

Previous results have indicated that a 15 s backward pairing between sucrose and odor elicits inhibitory conditioning [29], which is revealed through subsequent attempts to condition the PER using forward pairing (excitatory conditioning) to the same odor. The reported delay of 15 s corresponds to the time at which we observed olfactory interference, suggesting a relationship between olfactory interference and backward pairing induced inhibitory conditioning. Therefore, the animals from the previous experiment (Figure 3) were trained using a single forward pairing conditioning trial one hour after the olfactory interference protocol. The training consisted of presenting the odor used during olfactory interference (a new odor in the “nothing” group) followed by feeding with sucrose. A retrieval test was performed 3 hours after this training to evaluate the performance of the bees. In this retrieval test, we recorded the proportion of bees responding to the odor. This protocol is summarized in Figure 4A, and the results can be seen in Figure 4B.

In order to try to find a relationship between the performance of the bees during the retrieval test and their behavior during the backward pairing, we defined a behavioral factor corresponding to different types of response during backward pairing. Levels of this behavioral factor are presented in Figure 4A. We analyzed the performance during the retrieval test by using a forward stepwise logistic regression with three factors: odor (1-nonanol or octanal), foraging group (nectar or pollen), the behavioral factor and all of the possible interactions between factors. The stepwise procedure only kept one significant variable: the behavior factor (4 degrees of freedom χ^2^ = 24.832, p = 5.4 * 10^−5^). Figure 4B clearly illustrates that the performance of the group that retracts the proboscis early as well as the performance of the olfactory interference group are lower than in the group in which the proboscis was extended beyond 19 s (respectively 1 degree of freedom χ^2^ = 24.820, p = 6.3* 10^−7^ and χ^2^ = 6.148, p = 0.013). This indicates that olfactory interference disrupts the benefits of having a long lasting (>15 s) PER after sucrose feeding. Through interruption of proboscis extension, olfactory interference impaired subsequent learning, probably by forming an inhibitory association between the PER and the odor. This latter result is consistent with a previous report showing that following a 15 s backward pairing honey bees learn poorly relative to controls when given excitatory conditioning with the same CS [29]. In regard to our work, the major finding from this experiment is that olfactory interference is linked to backward inhibitory conditioning and is, as we will argue below, a kind of “inhibitory sensitization”.

However, this result does not specify whether olfactory interference generates a specific inhibition concerning only the odor that elicited it, or whether olfactory interference is unspecific (the bee learns to be unresponsive whatever the odor). To address this question, we used nectar foragers to perform a 15 s backward pairing with either 1-nonanol or octanal, as in our olfactory interference experiments above. This treatment was followed one hour later by two forward pairing trials, one of each with the olfactory interference odor and the other one with the other odor (trials were in random order and separated by 15 minutes). This conditioning was followed by a retrieval test for both odors 3 hours after. If olfactory interference inhibition is specific to the odor that was used during backward pairing, then the performance toward the novel odor should exceed that to the backward paired odor.

**Figure 5:**
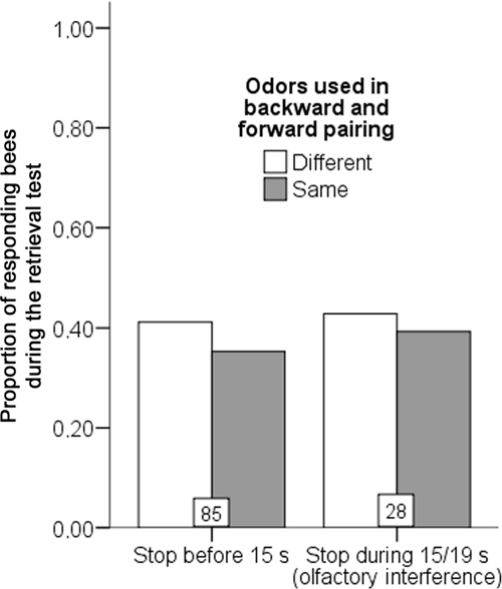
Effect of a backward pairing with one odor on the response to this odor and another odor. Performance of the bees during a retrieval test. This test was performed 3 hours after a single trial with each of two odors, one having been used in a previous backward pairing for olfactory interference (“same”) and the other being a novel odor (“different”). The bars are presented as a function of the behavior of the bees during the backward pairing. The boxes at the base of each bar indicate the sample size. Three bees only extended their proboscis beyond 19 seconds, so they are not represented here.

The results are presented Figure 5. We examined the treatment effect on three factors: the odor used during backward pairing (1-nonanol or octanal), the behavior of the bee during the backward pairing (retracted its proboscis before, during or after the odor presentation) and whether the odor had been presented during backward pairing or was the novel odor (this factor is a within individual measurement). Neither these three factors nor their interactions had a significant effect (generalized estimating equations, p>0.050 in all cases). This means that the bees did not discriminate between the odor that had been pre exposed and other odor. As a result, olfactory interference is not specific to the odor with which it was conducted. In summary, inhibitory conditioning is correlated to olfactory interference. However, this inhibition is not odor specific and rather affects the tendency of the bee to release a PER.

### Cimetidine, histamine and sucrose sensitivity

In a first attempt to unravel the potential role of inhibitory receptors in olfactory interference, we assessed the effect of potential antagonists and agonists of histamine receptors on backward pairing (see materials and methods for details); however, we cannot exclude that they target other types of receptors, because their specific effects are unknown in bees. We first examined the effect of injection of drugs or saline into the deutocerebrum on sensitivity to sucrose stimulation. Injection into the deutocerebrum affects two distinct areas: the antennal lobe (the first relay of olfactory information in the insect brain [56], [57]) and the dorsal lobe (this structure receives gustatory and mechanosensory information from the antennae [58]–[60]). Histamine and other inhibitory neurotransmitters have been shown to affect inhibition in the antennal lobe during the processing of olfactory information in the honeybee [33], [37], [48], [49]. Furthermore, the antennal lobe also plays a role in olfactory plasticity [30], [40], [61]–[66]. We therefore hypothesized that the drugs we used could affect olfactory interference and/or olfactory pathways.

**Figure 6:**
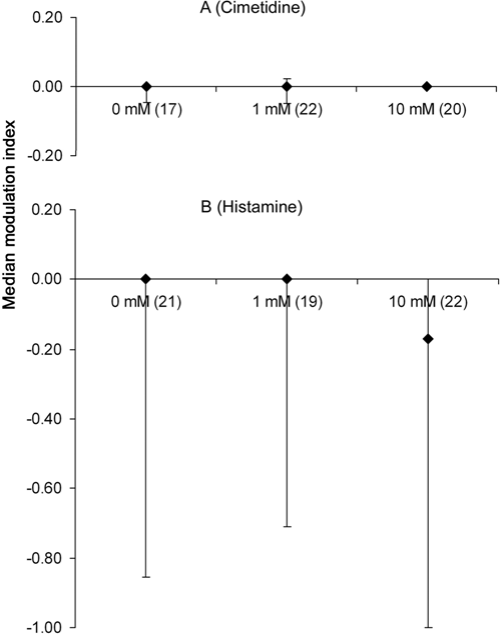
Effect of histaminergic drugs on sucrose sensitivity modulation index. (A) The median (50^th^ quantile) sucrose sensitivity modulation index (MI) for a range of concentrations of cimetidine. The modulation index corresponds to the variation that occurs in sucrose sensitivity before and after treatment (see methods). Error bars are the interquartile interval, i.e. interval between 25^th^ and 75^th^ quantiles. Note that when the 25^th^ and/or the 75^th^ quantile have the same value than the median there is no error bar to display (e.g. group cimetidine 10 mM). Numbers in parenthesis are the sample size. (B) The sucrose sensitivity modulation index for bees treated with histamine. Data are shown in the same manner as in A.

Sucrose sensitivity was assessed before and after injection into the deutocerebrum with either cimetidine (antagonist; 1 or 10 mM) or histamine (agonist; 1 or 10 mM); saline alone (0 mM) served as the control. A modulation index (MI, see materials and methods and [42]) was computed for each bee. The MI evaluates changes in sucrose sensitivity as a result of, in our case, pharmacological treatment. Neither 1 mM nor 10 mM of cimetidine (Figure 6A) or histamine (Figure 6B) had a significant effect on sensitivity when injected in the deutocerebrum (cimetidine, Kruskall-Wallis test: χ^2^ = 1.743, 2 degrees of freedom, p = 0.418; histamine, Kruskall-Wallis test: χ^2^ = 1.046, 2 degrees of freedom, p = 0.593). In all cases the MI was equal to or close to zero. Experiments conducted with histamine led to a wider range of MI values (compare the interquartile interval in Figure 6A and 6B), but this effect is controlled by having a saline group specific to each experiment. Since the same range is apparent in the control group, it is unlikely to be due to an effect of histamine injection. Instead, it was probably a result of seasonal- or weather-related variation in bee physiology [67]–[69]. Furthermore, after the injection PER release for the highest sucrose concentration (30%) was similar across the three groups in both experiments (data not shown; cimetidine, Fisher's exact test: p = 0.568; histamine: χ^2^ = 0.663, 2 degrees of freedom, p = 0.718). In conclusion, histamine and cimetidine did not affect sucrose sensitivity or proboscis extension.

### Effect of cimetidine on olfactory interference

**Figure 7 pone-0003513-g007:**
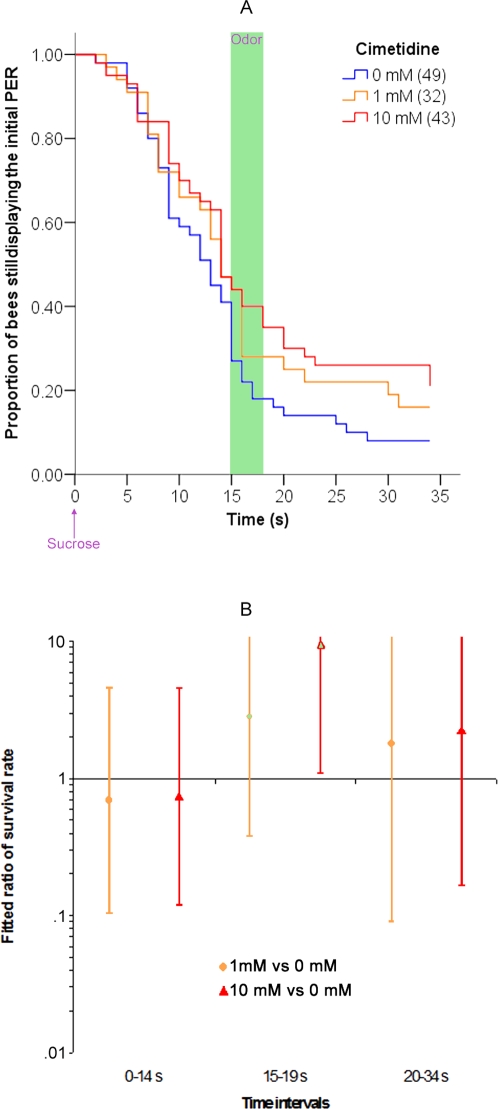
Effect of cimetidine on olfactory interference. (A) Proportion of bees that are still releasing the initial PER as a function of the time after the onset of the sucrose feeding. There were three groups of bees: animals injected into the deutocerebrum with 0 mM (control), with 1 mM or with 10 mM of cimetidine 15 minutes before starting the experiment. Other details are as in Figure 1A. (B) Ratio of the “survival rate” of the PER in the Cox regression for bees treated with 0 mM, 1 mM or 10 mM of cimetidine. The data are presented as in figure 1B.

Fifteen minutes before starting the olfactory interference experiment (conducted as above), cimetidine (either 1 or 10 mM) or saline control (0 mM) was injected into the deutocerebrum. The results are reported in Figure 7. Treatment with cimetidine showed a significant impairment of olfactory interference. There was no effect of cimetidine on performance before the odor was delivered (before the odor, 0–14 s, Wald test; 0 mM vs. 1 mM: χ^2^ = 0.143, p = 0.705; 0 mM vs. 10 mM: χ^2^ = 0.106, p = 0.744), but the 10 mM cimetidine group was significantly different from the saline group once the odor was delivered (Wald test; 0 mM vs. 10 mM during the odor, 15–19 s: χ^2^ = 4.169, p = 0.041). Bees in the 10 mM group failed to show the expected decrease in the rate of PER (i.e. there was no olfactory interference). The response of the 1 mM cimetidine group was intermediate between that of the 10 mM and saline groups, but it was not significantly different from the saline group (Wald test; 0 mM vs. 1 mM during the odor, 15–19 s: χ^2^ = 1.015, p = 0.314). After the odor, the behavior of the drug-treated groups were identical to that of the saline group (Wald test after the odor, 20–34 s; 0 mM vs. 1 mM: χ^2^ = 0.150, p = 0.699; 0 mM vs. 10 mM: χ^2^ = 0.373, p = 0.541).

**Figure 8:**
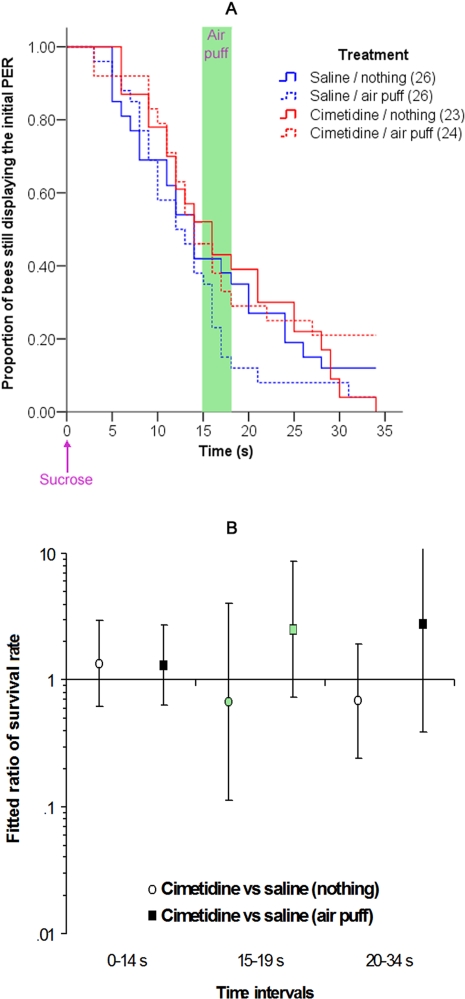
Control for the specificity of cimetidine effect. (A) Proportion of bees treated with 0 or 10 mM cimetidine that continue to release the initial PER as a function of time when presented with nothing or an air puff 15 s after sucrose presentation. Other details are as in Figure 1A. (B) Ratio of the “survival rate” of the PER in the Cox regression for bees treated with 0 mM or 10 mM of cimetidine. The data are presented as in figure 1B.

We performed the following control experiments to rule out other interpretations of the pharmacological effects. The spatial specificity of the injection was confirmed by injecting cimetidine in the lobula of the optic lobe, instead of into the deutocerebrum. The lobula is not involved in chemosensory processing or olfactory learning and is a standard control for the spatial specificity of treatments in bee olfactory learning [62], [63], [70]. If the drug spreads from the injection site to other brain areas, then we could not localize the effect to the deuterocerebrum and injection into the lobula would have the same effect. However, injection into the lobula had no effect on olfactory interference (supplementary Figure S5). Similarly, we injected into the deutocerebrum either cimetidine (10 mM) or saline 15 minutes before presenting sucrose to the bees, but this time either an air puff or no stimulation at all was presented instead of the odor (Figure 8). In this situation, the respective cimetidine- and the saline-treated groups (comparison of both solid lines or both dashed lines in Figure 8) did not significantly differ at any time-period (Wald test, p>0.050), ruling out the possibility that cimetidine enhances expression of the PER. Interestingly, presenting an air puff to the saline-treated bees elicited a significant decrease of the PER, even though no odor was presented (comparison of blue solid and dashed lines in Figure 8). There was a significant difference between untreated bees and bees exposed to air puff at the 15–19 s time period (Wald test, χ^2^ = 4.620, p = 0.032), whereas these two groups did not differ at other time periods (0–15 s, χ^2^ = 0.070, p = 0.792; 20–34 s, χ^2^ = 0.119, p = 0.731). Hence, there could be a small “mechanosensory interference” effect as well, although it is not very consistent (Figure 1).

We conclude that cimetidine starts to impair olfactory interference at a dose between 1 mM and 10 mM (Figure 7). This effect cannot be explained by an effect of cimetidine on sucrose sensitivity (Figure 6A), nor on the course of the ongoing PER (Figure 8). Thus, cimetidine blocks odor perception and/or olfactory interference itself. Interestingly, pyrilamine had an effect on olfactory interference similar to that of cimetidine (see supplementary Figure S6).

### Effect of histamine on olfactory interference

**Figure 9:**
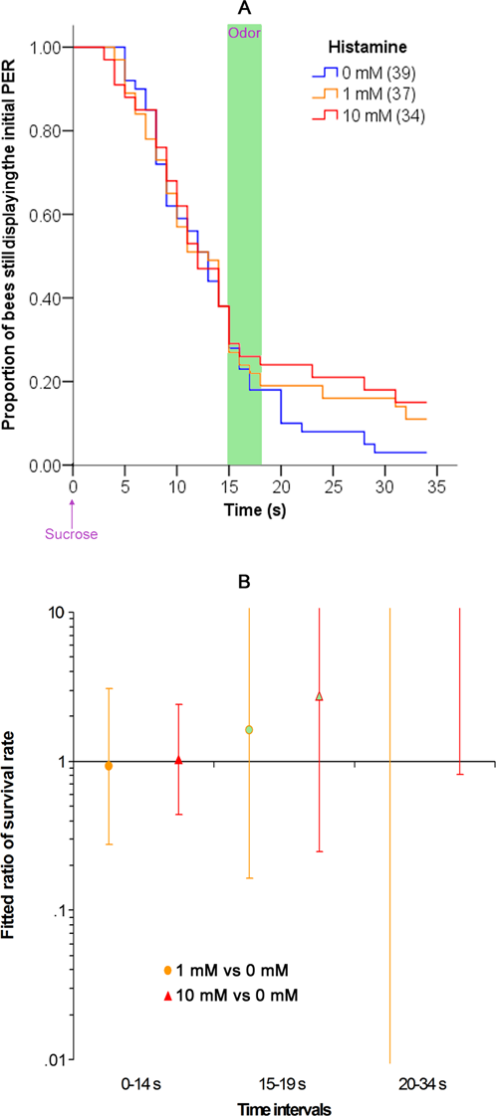
Effect of histamine on olfactory interference. (A) Proportion of bees that are still releasing the initial PER as a function of the time after the onset of the sucrose feeding. There were three groups of bees: animals injected into the deutocerebrum with 0 mM (control), with 1 mM or with 10 mM of histamine 15 minutes before starting the experiment. The odor (either 1-nonanol or octanal) were presented 15 s after the onset of the sucrose feeding, during 4 s. Other details are as in Figure 1A. (B) Ratio of the “survival rate” of the PER in the Cox regression for bees treated with 0 mM, 1 mM or 10 mM of cimetidine. The data are presented as in figure 1B.

Histamine (1 or 10 mM) was injected to bees using saline (0 mM) as control, in a way identical to the experiment with cimetidine. Results are reported in Figure 9. There was no difference between the treated groups and the control group at any time, including during the odor presentation (Wald test: p>0.050 in all cases). This indicates that injection of exogenous histamine does not affect olfactory interference. Although cimetidine impaired olfactory interference, this does not necessary mean that histamine should enhance it. Indeed, it is possible that the histaminergic system in the deutocerebrum is already acting close to one end of its dynamic range. In this case, adding more histamine would not have an effect, whereas addition of the histamine receptor antagonist cimetidine would have measurable consequences. Alternatively, the effect of cimetidine might be unrelated to histamine receptors, especially because the effective dose (10 mM) is quite high.

## Discussion

### Olfactory interference

The main finding of our work is the observation of olfactory interference, previously unreported for honey bees. We have found that in olfactory interference, presentation of an odor stimulus 15 sec after sucrose feeding terminates an on-going PER (i.e. it desensitizes the bee). Moreover, presentation of odor earlier (7 sec) or later (30 sec) did not interfere with the ongoing PER. A similar phenomenon was previously reported by Dethier [54] in blow flies. When flies feed on a droplet of sucrose solution they respond appetitively for several seconds after feeding. Dethier reported that this sensitization could be ‘discharged’ by application of a water droplet to the mouthparts; however it is unclear whether this discharge effect resulted from a sensory or more central process. Olfactory interference in the honey bee is likely due to a central process, because it involves the interaction of taste, PER release and olfaction; it is unlikely that an odor stimulus would interfere with PER release or taste at a peripheral level.

### Olfactory interference and inhibitory conditioning

Backward presentation of US and CS (i.e. the reverse of forward pairing that produces excitatory conditioning) inhibits subsequent conditioning with this CS [71]–[73]. Previous work with honey bees reported that backward pairing with a specific delay of 15 s produces inhibitory conditioning [29]. Similar backward inhibitory conditioning has been reported for aversive learning in fruit fly [74] (see also [75] for a study of backward pairing of various odors in bees). We show that olfactory interference is related to backward pairing based inhibitory conditioning in a way that is analogous to the relationship between sensitization and excitatory conditioning [15], [16]. In other words, forward excitatory conditioning is initially related to sensitization, whereas backward inhibitory conditioning is initially related to olfactory interference. Curiously, the only paper on backward pairing of odor and sucrose in honey bees [29] did not report olfactory interference. Inhibitory conditioning induced by backward pairing [29] and olfactory interference both are maximal at 15 s after sucrose presentation. Furthermore, shortened PER durations induced by olfactory interference are correlated to a lower performance on subsequent excitatory conditioning. This means that olfactory interference and inhibitory conditioning probably share, at least in part, some underlying mechanisms. Therefore, it seems likely that the neural and biochemical basis for olfactory interference is engaged for inhibitory conditioning, just as the neural and biochemical machinery for sensitization is engaged to drive excitatory conditioning [15], [16].

Interestingly, pairing the same stimuli (in our case, sucrose and odor) can lead to both excitatory and inhibitory conditioning depending on to the way the pairing is made. The decisive parameter for switching between excitation and inhibition is the timing of the pairing of odor and sucrose: forward pairing will produce excitatory learning, whereas backward pairing will lead to inhibitory conditioning through olfactory interference. Further work is needed to completely explore this effect, for example testing the effect of repeated backward pairing (to see if olfactory interference is facilitated and if subsequent learning becomes even more inhibited) or using various time delays between backward pairing and forward pairing (thus testing the duration of the inhibitory trace formed during backward pairing).

The air stimulus (a mechanosensory stimulation) carrying the odor might also elicit interference with the ongoing PER (Figure 8), although our results established that odor was more salient in causing the proboscis retraction (Figure 1). This suggests that antennal tactile [34], [39], [76]–[79] and antennal mechanosensory [80] conditioning could also be used in backward pairing with sucrose. We therefore predict the occurrence of a tactile/mechanosensory interference, as well as inhibitory tactile/mechanosensory conditioning. Moreover, olfactory aversive conditioning can also be done in laboratory conditions [81], [82]. The aversive conditioning paradigm rests upon extension of the sting elicited by an electric shock; several pairings of an odor with an electric shock condition the bee so that subsequent presentations of the same odor elicit extension of the sting. Olfactory interference and backward inhibitory learning could also occur with this protocol.

Finally, the main interest of olfactory interference is that it links the odor and the PER (CS/response association). Following the work of Hammer [14], [40], [64], most studies of the neural basis of olfactory conditioning have focused on finding central sites of convergence between olfactory and sucrose stimuli (CS/US association, [13], [14], [16]). However, during Pavlovian conditioning the CS/US association is not the only one to occur and CS/response association can also be elicited [83]. As olfactory interference is based on an interaction between PER and olfactory perception, understanding the basis of olfactory interference may shed new light on the basis of olfactory conditioning by providing the possibility of studying the interaction between PER and odor.

### Pharmacological and neuroanatomical aspects

Further investigations of olfactory interference may establish the biochemical basis for its time course. We have reported our first investigation of the neural network basis of olfactory interference by injecting cimetidine into specific brain regions. In fruit flies, cimetidine is an antagonist of histamine receptors [84]–[86]. Because of the similarity between fruit fly and honey bee histamine receptors [84], cimetidine might block the histamine receptors in bees as well. However, in the honey bee the targets for cimetidine and the histamine receptors have not been characterized, so it is too early to specifically conclude that histamine receptors are involved in olfactory interference or olfactory perception. A reasonable, somewhat more conservative, conclusion is that cimetidine-sensitive receptors are involved in olfactory processing that gives rise to olfactory interference [33], [37], [48], [49], [88]. The effects of other drugs were not significant.

We made injections into the deutocerebrum, which includes the antennal lobes. Antennal lobes are analogs to the mammalian olfactory bulb as they are the first-order synaptic process of olfactory information [56], [57]. However, the antennal lobe is close to the dorsal lobe (together they form the deutocerebrum). Because of this proximity, it is likely that injection made in the antennal lobe affected the dorsal lobe as well. This is relevant because the dorsal lobe receives gustatory information from the antennae [59], and thus could also be involved in olfactory interference. As a localization control we injected cimetidine into the lobula of the optic lobe. If the drug had spread broadly to many areas of the brain, then injection into the optic lobe should have produced the same effect as injection into the antennal deutocerebrum. Failure to find this effect substantiates our argument that olfactory interference involves an interaction between olfactory and taste modalities in the deuterocerebrum.

As the antennal lobe is also the first relay of olfactory information, one explanation for the effect of an injection of cimetidine into the deutocerebrum could be that this drug blocks or modifies the perception of odor. In particular, this explanation is consistent with results previously obtained in insects that indicated that when inhibition is altered by injection of histamine, bicuculine or picrotoxin in the antennal lobe, it results in greatly increased odor detection thresholds [49], [88]. As a result, two hypotheses can be proposed for the neural substrate of olfactory interference. In the first one, olfactory interference is driven by the output of the antennal lobe, which affects the motor integration centers that control PER. In this case, the output of the antennal lobe would be either permissive (during olfactory conditioning) or repressive of the PER (during olfactory interference) depending on when odor is detected in the process of extending the proboscis. Cimetidine would block antennal lobe output, preventing the odor from affecting PER (probably because the odor is simply not detected). Alternatively, in the second hypothesis, the antennal lobe would not have a direct role in olfactory interference and would simply relay olfactory information; cimetidine would affect or block this relay. In this case, another part of the brain would gate the PER release as a function of olfactory information (according to the timing, the presence of an odor would switch the system to PER repression in olfactory interference or to PER release after olfactory conditioning).

A candidate for the location of the motor control of the PER is the protocerebral lobe. Indeed, previous results have suggested that this brain region could be a pre-motor area for the PER release [89]. More specifically, an inhibitory system would block the PER, and this inhibitory system would be activated by odor presentation during olfactory interference but deactivated after olfactory learning (from Figure 7 in [89]). To investigate these hypotheses further, calcium imaging [56] and electrophysiology [37] may be used in conjunction with pharmacology [49].

## Materials and Methods

### Animals

Honey bees workers (*Apis mellifera*) were standardized in regard to foraging task. Bees were collected in front of the hive entrance by capturing non-pollen foragers in glass vials as they returned to the colony. Pollen foragers can be identified by the pollen load carried on the hind legs, and for most experiments they were excluded because their sensitivity to sucrose differs from non-pollen foragers [55]. Furthermore, pollen foragers are less common, particularly at certain times of the year, whereas non-pollen foragers are readily available. However, in some experiments pollen foragers were used for comparison purposes (see results).

The bees were immobilized by leaving the vials on ice. Animals were then restrained in small metal tube with strips of tape on the abdomen and behind the head. Once they recovered from chilling, they were fed 15 µl of 1.17 M sucrose solution and kept overnight in a humidified box until they were used for experiments the next morning. When the animals were to be injected, the head was immobilized by a small drop of orthodontic sticky wax before feeding. In order to prepare them for injection (see below), a window was cut in the cuticle above the antennae and between the eyes immediately after the preparation of all the bees. The antennal lobes were then revealed by gently pushing aside the glands and the white tracheal sheath. The survival rate after the operation varied between 30% and 75%, according to the season.

### Behavioral procedures: olfactory interference

All experiments started by presenting 0.6 µl of 1.17 M sucrose solution to both antennae and then to the proboscis, except for one experiment when the effect of presenting the sucrose to antennae only was investigated (supplementary Figure S3). The bees were allowed time to drink all of the solution (i.e. a few seconds), which ensured that all bees had consumed the same amount of sucrose. Under normal circumstances, the proboscis can remain extended and appetitive feeding movements may continue after the sucrose droplet disappears. We evaluated the effect of odor presentation on this ongoing response by presenting odor for 4 s beginning at three different points after feeding onset (defined at the moment when the tip of the proboscis touches the sucrose solution droplet). In different experiments, delays of 7 s, 15 s or 30 s were used. When bees were treated with a drug before the experiment (see below), the injection took place 15 minutes before the sucrose stimulation.

In some experiments, bees were also trained using olfactory conditioning. A conditioning trial consisted of presenting an odor for 4 s, and 3 s after the onset of the odor 0.6 µl of 1.17 M sucrose solution was presented to both antennae and then to the proboscis. The bees consumed the sucrose solution. Three hours after conditioning, bees were tested (retrieval test) by presenting the same odor without reinforcement and the response was recorded. If the bee did not release a PER to the odor during this retrieval test, the sucrose solution was presented again; if the bee failed to respond to the sucrose, then its performance was not taken into account.

The odor was delivered by an electronically controlled device that directed an air flow (ca 1450 ml/min) through a syringe containing a strip of filter paper bearing 3 µl of the pure odorant. The odor strips were freshly prepared for each experiment. The syringe remained 2 cm in front of the platform on which the bees were placed for the whole experiment. The platform was positioned in front of an exhaust hood to prevent odorant accumulation after delivery of the odor. Odors used were 1-nonanol or octanal (both from VWR).

Sucrose feeding led to a long-lasting PER that often continued even after the bee had finished drinking the sucrose solution. We measured the PER initial duration, defined as the duration of the PER after sucrose feeding onset. The performance of the bees was videotaped for 35 s after the feeding onset, and for each second we recorded whether the initial PER was still occurring (i.e. analyses were always done in discreet time). Bees would sometimes briefly retract their proboscis before resuming the PER; retractions of one second or less were not considered to interrupt the initial PER. In theory, bees that retract their proboscis could restart to release a PER when odor is presented if they are sensitized. However, we hardly ever observed that a bee that stopped releasing the initial PER for more than one second would resume it, whether or not an odor was presented. Thus, the total time spent displaying the PER is strongly correlated to this initial duration (see supplementary Figure S1 for comparison of the two values and a detailed discussion of this aspect).

### Behavioral procedures: sucrose sensitivity

Sucrose sensitivity is an important parameter correlated with many honey bee behaviors [90] and the effects of drugs on sucrose sensitivity can be assessed using the protocol developed by Scheiner et al. [42]. An important first step before using drugs in olfactory interference was to check the potential of effect of these drugs on sucrose sensitivity. A series of six sucrose solutions of various concentrations was presented in random order (0.1%, 0.3%, 1%, 3%, 10% and 30%, with 10% = 100 g.l^−1^ or 29 mM; this corresponds to an increasing logarithmic series: −1, −0.5, 0, 0.5, 1, 1.5). For each one of these solutions, both antennae were touched by a 0.6 µl drop of sucrose solution and the occurrence of PER was recorded; animals were not fed with the sucrose to prevent satiation effects. There was a delay of 2 minutes between each concentration. To avoid sensitization or habituation of the animals to repeated sucrose presentation, a 0.6 µl drop of distilled water was presented to both antennae between each sucrose presentation (1 minute before the next sucrose presentation). Furthermore, an interval of 2 minutes between sucrose presentations does not promote habituation to sucrose [91].

After the first presentation of the whole series, animals received an injection of saline (vehicle of the drugs, see below) or drug (histamine or cimetidine, 1 or 10 mM). All sucrose solutions were then presented as previously 15 minutes after the injection. For each bee, we recorded the number of PERs during the presentation of the sucrose solution series before (PRE) and after (POST) the injection; as there were six sucrose solutions presented, PRE and POST scores ranged from 0 to 6. We computed for each bee a modulation index (MI) according to Scheiner et al. [42]: MI = (POST−PRE)/(POST+PRE). MI varies from −1 (for a bee responding to sucrose before injection and stopping doing so after the injection) to 1 (for a bee not responding to any sucrose concentration before the injection and starting doing so after the injection). A value of 0 denotes the same performance after and before the injection. When both PRE and POST were equal to 0, MI was defined as 0 (no change).

### Drugs and injection

Drugs were injected into both antennal lobes. However, it is likely that an injection in the antennal lobe affects the whole deutocerebrum, i.e. the antennal lobe plus the dorsal lobe. Therefore, to be conservative we used the term “injection into the deutocerebrum” throughout the text. The properties of histamine receptors in the deutocerebrum of adult honey bees are not known. Therefore, we chose drugs according to their effects on fruit fly histamine receptors [84]–[86], as they are similar in molecular structure to receptors in the honey bee and conserved across different insects [87], [92]. The drugs used (all from Tocris) were histamine (agonist of histamine receptors), cimetidine, pyrilamine ( = mepyramine) and dimaprit (in fruit fly, the last three are antagonists of histamine receptors; the results for pyrilamine and dimaprit are used in supplementary Figure S6). All the drugs were dissolved in a bee phosphate buffered saline solution which was also used for control injection (pH 7.8, 609 mosmol.l^−1^: 2.7 mM KCl, 154.0 mM NaCl, 1.8 mM CaCl_2_, 11.7 mM sucrose, 80.5 mM Na_2_HPO_4_, 18.5 mM NaH_2_PO_4_).

The solutions to be injected were loaded into a glass microelectrode connected to a microinjector (picospritzer II, Parker Hannifin Instrumentation). These microelectrodes were made by pulling quartz glass capillaries (outer diameter 1 mm, inner diameter 0.5 mm) with a microelectrode puller (P2000, Sutter Instrument Company). Then the pulled, unopened tip of each capillary was gently broken. The injection volume was calibrated by measuring with a graduated ocular the size of drops injected in mineral oil. Each deutocerebrum received a volume of 4.2 nl. Immediately after the injections, the injected volume was checked again, and if variation had occurred the bee was not used.

### Data treatment

Unless otherwise mentioned, statistical analyses were performed with SPSS 15.0 (SPSS Science). All the tests were two-tailed. Statistics were evaluated using an α risk of 0.050. For sucrose sensitivity experiments, we compared the MI across the groups using the Kruskall-Wallis test, as this index is not distributed normally. We also compared the proportion of honey bees releasing a PER following the injection for the 30% sucrose solution, using χ^2^ tests, or Fisher's exact test (computed with R 2.4, http://www.r-project.org/ [93]) if the χ^2^ test assumptions were not met. In the conditioning experiment, we used the generalized linear model (forward stepwise logistic regression or generalized estimating equation) to assess the role on the performance of different factors.

For olfactory interference experiments, we used time-dependent Cox regression (also known as proportional hazard survival analysis, procedure COXREG in SPSS; see [94]) to compare the initial duration of the PER between different groups (that is, the “survival” of the PER across the time). This technique is especially designed to treat this type of data, and it allows us to take into account the few bees that were still releasing a PER at the end of the behavioral monitoring. We used the time-dependent version of Cox regression in order to consider the different time-periods: before odor onset, during and one second after the 4 s odor presentation and after the odor presentation. As there were usually few bees still extending their proboscis at the end of the recording period, it sometimes happened that the Cox regression could not yield p-values for these periods; this meant that there was not enough animals still displaying a PER to observe a relevant effect.

It is important to understand that the Cox regression compares across treatment groups the probability (‘risk’ in regard to the terminology of Cox regression) of terminating PER during a given time period; or equivalently, the “survival rate” of the PER in the considered time-period. This is better and more convenient than other types of statistical analyses (e.g. χ^2^) that would have repeatedly compared the proportion of animals exhibiting PER at each second and is more specific than comparing the PER duration with an ANOVA (which ignores the time-period).

We defined contrast tests for each of the time-periods (before, during and after the odor) to compare the probability of PER termination between treatment groups (in other words, the “survival” of the PER). The statistical significance of these contrasts was evaluated with Wald test (distributed like a 1 degree of freedom χ^2^). As contrasts are required to be orthogonal from each other, only a limited number of tests could be done. Thus, we could only compare each drug concentration (or each treatment) to the corresponding control group as supplementary contrasts would not have been independent. For all experiments, the performance of the bees was similar for both odors (1-nonanol vs. octanal within each group, before, during and after odor presentation: Wald test, p≥0.050 in all cases), so we pooled the results of the two odors (octane and 1-nonanol) in all figures and analysis (except in Figure 1 where results for the two odors are shown separately as an example).

Each contrast (e.g. control vs. treated) corresponds to the fitted ratio of survival rates in two groups, e.g. (fitted survival rate in treated)/(fitted survival rate in control). These values are plotted with their 95% confidence interval. When this confidence interval does not include 1, then the corresponding Wald test is significant, meaning that the survival rates are not the same in the considered period. For instance, if the ratio (fitted survival rate in treated)/(fitted survival rate in control) is lower than one during a given time period, this means that the bees retract their proboscis more often in the treated group than in the control group. These confidence intervals are reported in graphs along with the proportion of bees still displaying the initial PER as a function of time.

## Supporting Information

Figure S1 Comparison of results obtained when using initial duration and total duration of the PER. We analyzed the videotape of the initial olfactory interference experiment (Figure 1) to determine two values for each bee: (1) the initial duration of the PER, which is the time from the extension of the proboscis until it had been retracted for at least 1 s and (2) the total duration of the PER, that is the total number of seconds during which the bee had its proboscis extended over the 35 s recording period, even if the bee retract and re-extend its proboscis. Our analysis is based on the initial duration because it is conveniently analyzed by Cox regression and is related to sensitization. To determine if our choice of initial duration rather than total duration affected our results, we plotted the data from the experiment shown in Figure 1 (“initial” curve), but this time we added a second curve corresponding to the proportion of bees extending their proboscis at each second, whether it was the initial PER or not (i.e. the total duration). Therefore in this second “total” curve, a honey bee that stopped the PER and then resumed 2 or 3 s later was still included in the subsequent time periods. For each group in the first experiment (Figure 1), the proportion of bees releasing the initial PER and the proportion of bees releasing a PER (initial or not), which are respectively the “initial” and the “total” curves on each graph (the “initial” curve is identical to Figure 1). The sample size is given in parenthesis. Note that there is one animal less than in Figure 1 in the air treated group, because it was not recorded until the end of the 34 s due to the end of the tape (this allowed us to calculate its initial duration but not its total duration). In all cases, the curves for the initial and the total duration of PER are similar, which confirms that these two values are essentially the same. Furthermore, this analysis reveals that bees that stop releasing the initial PER usually do not resume it.
(0.98 MB TIF)

Figure S2 Replication of the olfactory interference experiment. As olfactory interference had not previously been described, we replicated our results for confirmation purposes. Shown are the proportion of bees displaying the initial PER versus time after sucrose. Details are as in Figure 1, except that there was no air-pulse treated group and that the data from the 1-nonanol and octanal groups are pooled. The odor group's probability of stopping the PER was similar to the control group's before and after the odor presentation (Wald test; before the odor, 0–14 s time-period: khi2 = 0.572, p = 0.450; after the odor, 20–34 s time-period: khi2 = 1.785, p = 0.181). On the other hand, there was a decrease of the proportion of bees still releasing a PER at the onset of the odor, which replicates the olfactory interference effect; as a result, the probability of stopping the PER release is significantly higher in the odor group when compared to the control group (Wald test; during the odor and 1 s after, 15–19 s time-period: khi2 = 5.349, p = 0.021).
(0.43 MB TIF)

Figure S3 Effect of presenting the sucrose to the antennae. In this experiment, we wanted to explore the effect of presenting sucrose on the antennae rather than on the proboscis. For antennal stimulation, the honey bees are not fed as they are with antennal+proboscis stimulation. Therefore, the olfactory interference protocol was replicated with three pollen forager groups: one was fed as usual and presented with the odor at 15 s after the onset of the feeding (control group), and the two others had the PER elicited by antennal stimulation but were not fed. These two groups were presented an odor at either 15 or 30 s after the antennal stimulation. However, stimulating the antennae did not elicit an enduring PER, making it impossible to evaluate olfactory interference. Moreover, the group receiving odor at 30 s did not display any sensitization-induced odor response PER.
(0.46 MB TIF)

Figure S4 Alternative representation of the data in Figure 4. In an alternative analysis of the data in Figure 4B, we used a forward stepwise logistic regression. We included the factors odor (1-nonanol, octanal), foraging (pollen, nectar), treatment (no odor, odor at 15 s, odor at 30 s), and all possible interaction terms. The forward stepwise procedure only kept two significant variables: foraging (1 degree of freedom khi2 = 7.449, p = 0,006) and treatment (1 degree of freedom khi2: nothing vs. 15 s, khi2 = 5.343, p = 0.021; but nothing vs. 30 s, khi2 = 0.056, p = 0.813). The odor factor and the two- and three-way interactions among the main factors were dropped during the stepwise procedure. This analysis indicates first that the two foraging groups (nectar and pollen) are different; in fact, as previously reported [17], [55], [90], [95]–[97] the pollen foragers overall performed better than the nectar foragers. Second, the 15 s group had a lower performance than groups that were either not treated or treated at 30 s. This corresponds to the group in which olfactory interference can be seen, so this is consistent with the analysis performed in Figure 4B. It also justifies our grouping of the “nothing” and “30 s” groups in Figure 4, as these groups are not different.
(0.64 MB TIF)

Figure S5 Effect of lobula injection of cimetidine on olfactory interference. To control the spatial specificity of the injection of cimetidine (Figure 6–8), the olfactory interference experiment was replicated except that injection of the drugs was into the lobula of the optic lobe instead of into the deutocerebrum. The lobula is not involved in chemosensory processing or olfactory learning and is a standard control for the spatial specificity of treatments in the bee learning [62], [63], [70]. We used cimetidine (10 mM) and saline (0 mM, control group), and the odor was 1-nonanol. All other details are as in Figure 7A. Contrary to what was seen in Figure 7, animals injected with cimetidine in the lobula were not different from animals injected with saline solution (Wald test; time-period 0–14 s: khi2 = 0.001, p = 0.971; time-period 15–19 s: khi2 = 0.183, p = 0.669; time-period 20–34 s: khi2 = 0.652, p = 0.419). This confirms that cimetidine does not diffuse beyond its target, although it may affect both antennal lobe and dorsal lobe when injected into the deutocerebrum.
(0.20 MB TIF)

Figure S6 Effect of dimaprit (A) and pyrilamine (B) on olfactory interference. To further investigate the effect of histaminergic drugs, two other histamine receptor antagonists (dimaprit and pyrilamine) were injected to honey bees following the same protocol. Other details are as in Figure 7A. (A) Animals that received 10 mM or 1 mM dimaprit were not significantly different from the corresponding saline group (Wald test, p>0.050 in all cases). This suggests that dimaprit is less efficient than cimetidine at impairing olfactory interference. However, olfactory interference is not very clear in this case. (B) For pyrilamine, after the odor was presented, the 10 mM and the 1 mM groups maintained the PER for a longer time than the control group, and showed a trend for reduced olfactory interference. This is similar to the effect of cimetidine. However, neither of the pyrilamine groups was significantly different from the control group (Wald test, p>0.050 in all cases; in particular, for 0 mM vs. 10 mM in the 15–19 s time period, khi2 = 0.542, p = 0.461). In the fruit fly, pyrilamine is more efficient than dimaprit in blocking histaminergic receptors made of HisCl2 subunits (IC50 of 165 and 279 µM respectively, cimetidine being at 117 µM) while the opposite effectiveness is seen for histaminergic receptors made of HisCl1 subunits (IC50 of 442 and 56 µM respectively, cimetidine being at 21 µM; all these data from [85]). Therefore, our results suggests that the receptors involved in impairing olfactory interference involve the AmelHisCl2 subunit, which is the ortholog of fruit fly dimaprit-insensitive HisCl2 subunit [87].
(0.46 MB TIF)

Movie S1 This movie illustrates the usual olfactory interference effect. A bee is presented with 0.6 µl of 1.17 M sucrose solution and is allowed to completely consume the sucrose droplet. This elicits a long-lasting PER. However, when an odor is presented 15 s after the onset of feeding, the bee immediately retracts its proboscis (in the movie the presence of the odor is signaled by the light).
(0.98 MB MPG)
