# Supplementary figures and images for: Olfactory Interference during Inhibitory Backward Pairing in Honey Bees
Source: PLoS One. 2008 Oct 23;3(10):e3513. doi: 10.1371/journal.pone.0003513 (PMC2568944; doi:10.1371/journal.pone.0003513)

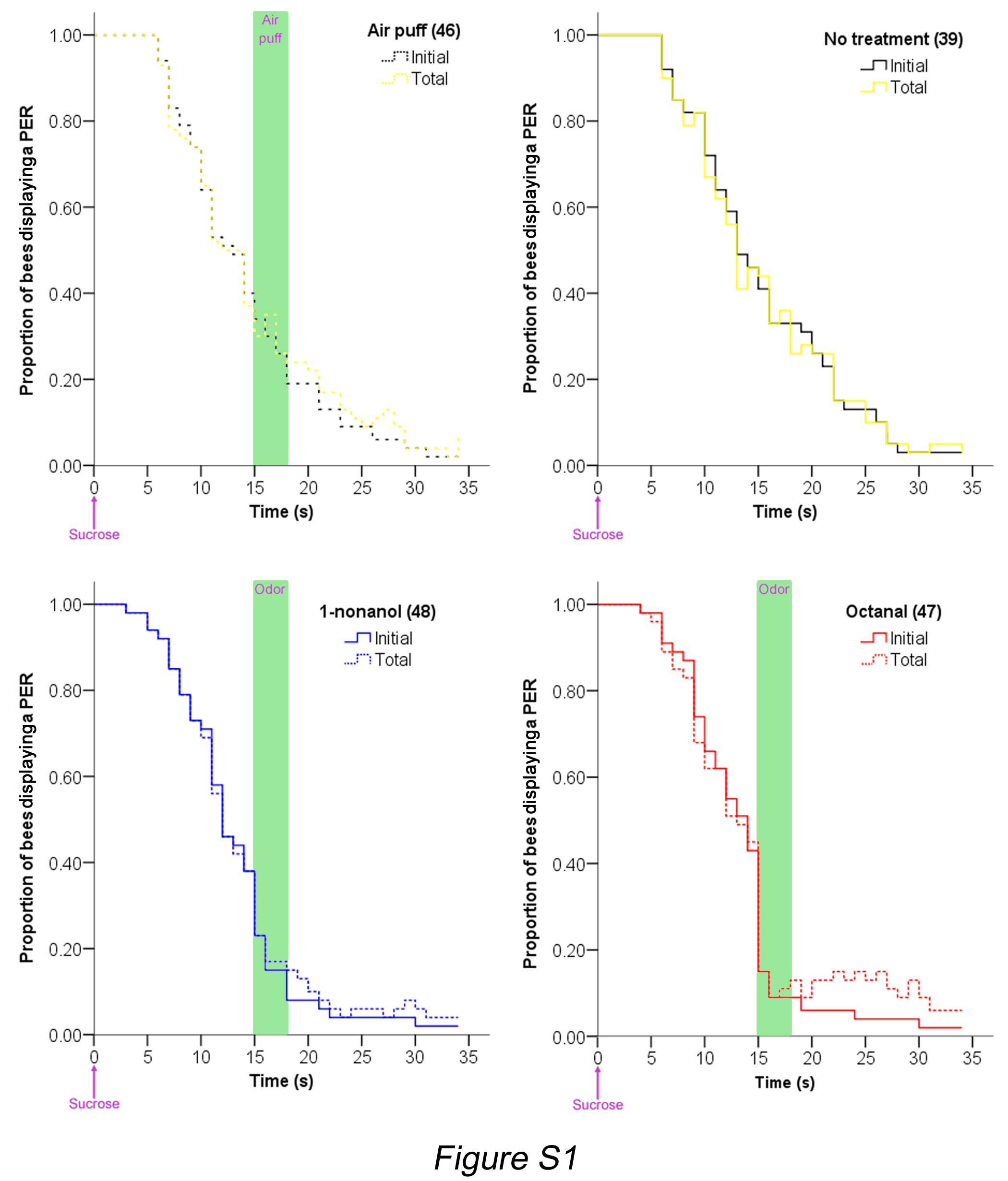

Supplement: Figure S1 — Comparison of results obtained when using initial duration and total duration of the PER. We analyzed the videotape of the initial olfactory interference experiment (Figure 1) to determine two values for each bee: (1) the initial duration of the PER, which is the time from the extension of the proboscis until it had been retracted for at least 1 s and (2) the total duration of the PER, that is the total number of seconds during which the bee had its proboscis extended over the 35 s recording period, even if the bee retract and re-extend its proboscis. Our analysis is based on the initial duration because it is conveniently analyzed by Cox regression and is related to sensitization. To determine if our choice of initial duration rather than total duration affected our results, we plotted the data from the experiment shown in Figure 1 (“initial” curve), but this time we added a second curve corresponding to the proportion of bees extending their proboscis at each second, whether it was the initial PER or not (i.e. the total duration). Therefore in this second “total” curve, a honey bee that stopped the PER and then resumed 2 or 3 s later was still included in the subsequent time periods. For each group in the first experiment (Figure 1), the proportion of bees releasing the initial PER and the proportion of bees releasing a PER (initial or not), which are respectively the “initial” and the “total” curves on each graph (the “initial” curve is identical to Figure 1). The sample size is given in parenthesis. Note that there is one animal less than in Figure 1 in the air treated group, because it was not recorded until the end of the 34 s due to the end of the tape (this allowed us to calculate its initial duration but not its total duration). In all cases, the curves for the initial and the total duration of PER are similar, which confirms that these two values are essentially the same. Furthermore, this analysis reveals that bees that stop releasing the initial PER usua [file pone.0003513.s001.tif]

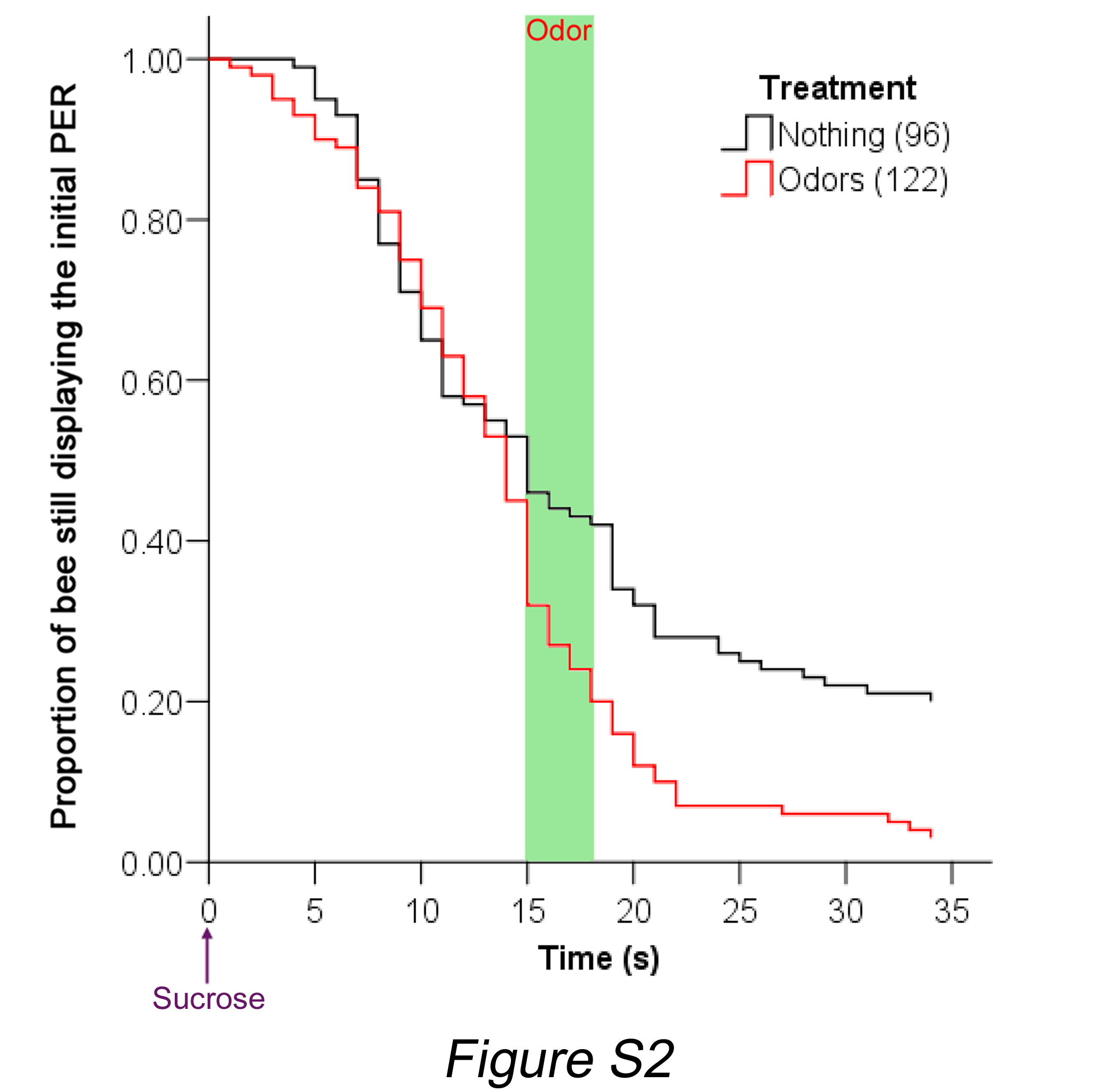

Supplement: Figure S2 — Replication of the olfactory interference experiment. As olfactory interference had not previously been described, we replicated our results for confirmation purposes. Shown are the proportion of bees displaying the initial PER versus time after sucrose. Details are as in Figure 1, except that there was no air-pulse treated group and that the data from the 1-nonanol and octanal groups are pooled. The odor group's probability of stopping the PER was similar to the control group's before and after the odor presentation (Wald test; before the odor, 0–14 s time-period: khi2 = 0.572, p = 0.450; after the odor, 20–34 s time-period: khi2 = 1.785, p = 0.181). On the other hand, there was a decrease of the proportion of bees still releasing a PER at the onset of the odor, which replicates the olfactory interference effect; as a result, the probability of stopping the PER release is significantly higher in the odor group when compared to the control group (Wald test; during the odor and 1 s after, 15–19 s time-period: khi2 = 5.349, p = 0.021). (0.43 MB TIF) [file pone.0003513.s002.tif]

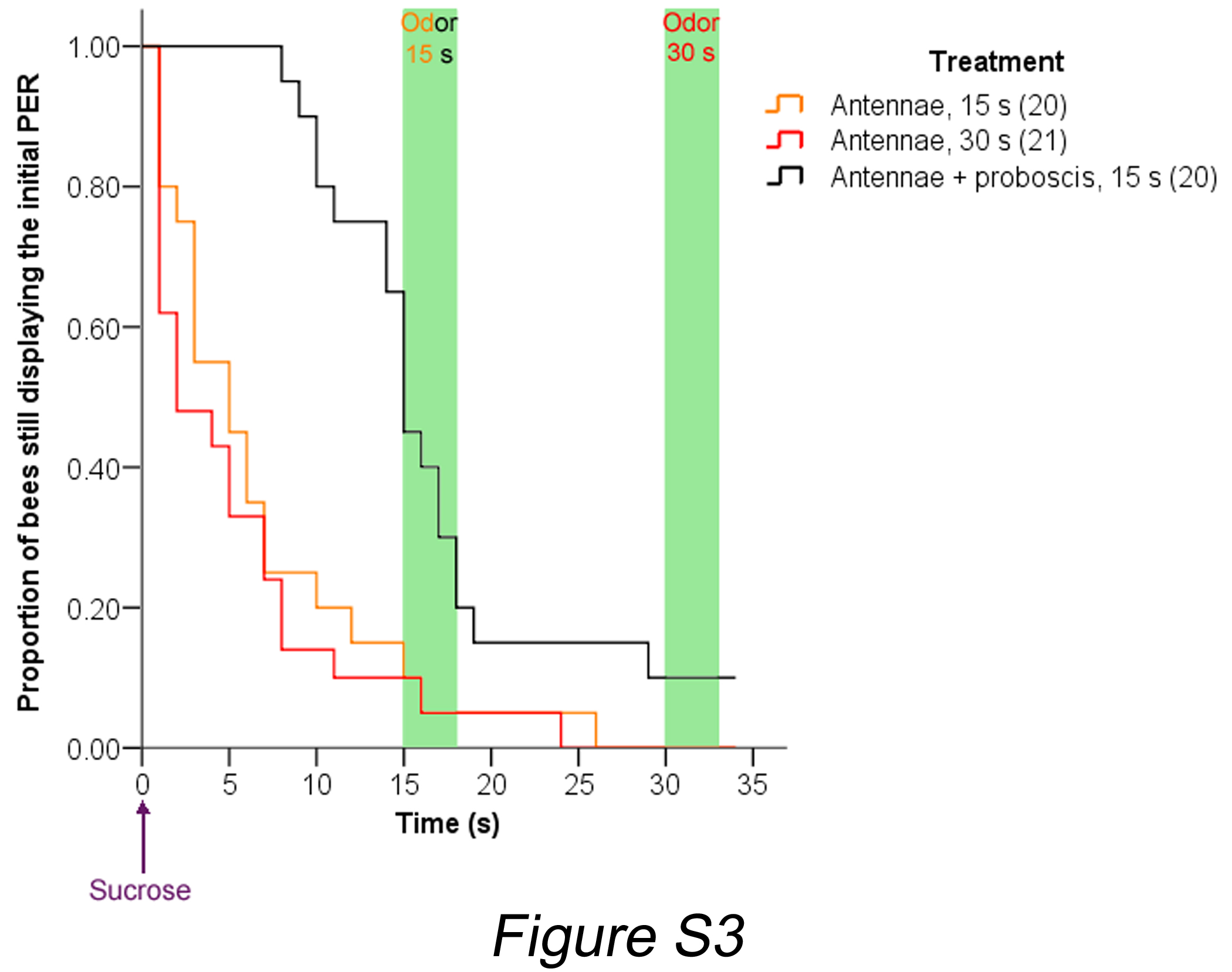

Supplement: Figure S3 — Effect of presenting the sucrose to the antennae. In this experiment, we wanted to explore the effect of presenting sucrose on the antennae rather than on the proboscis. For antennal stimulation, the honey bees are not fed as they are with antennal+proboscis stimulation. Therefore, the olfactory interference protocol was replicated with three pollen forager groups: one was fed as usual and presented with the odor at 15 s after the onset of the feeding (control group), and the two others had the PER elicited by antennal stimulation but were not fed. These two groups were presented an odor at either 15 or 30 s after the antennal stimulation. However, stimulating the antennae did not elicit an enduring PER, making it impossible to evaluate olfactory interference. Moreover, the group receiving odor at 30 s did not display any sensitization-induced odor response PER. (0.46 MB TIF) [file pone.0003513.s003.tif]

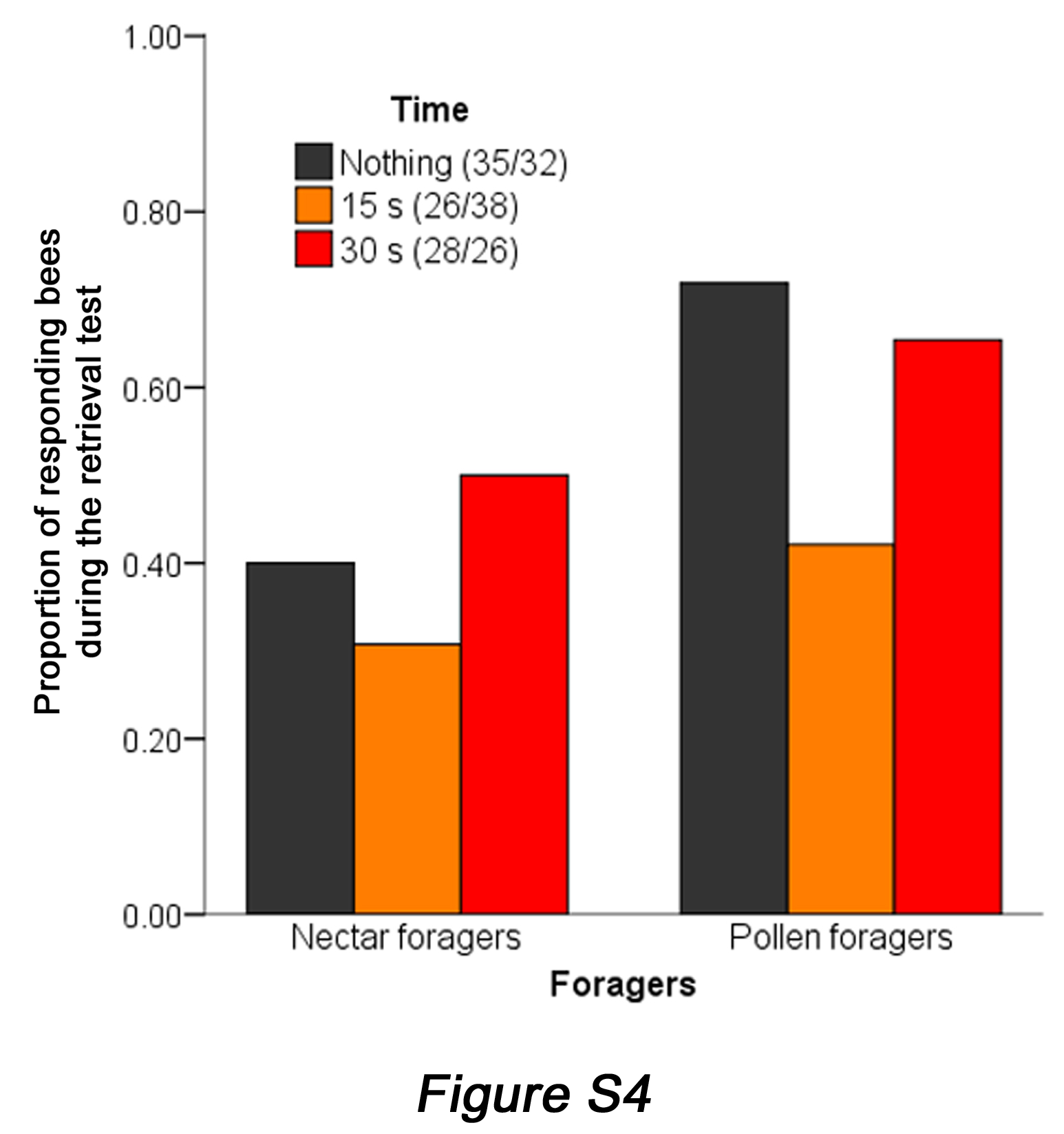

Supplement: Figure S4 — Alternative representation of the data in Figure 4. In an alternative analysis of the data in Figure 4B, we used a forward stepwise logistic regression. We included the factors odor (1-nonanol, octanal), foraging (pollen, nectar), treatment (no odor, odor at 15 s, odor at 30 s), and all possible interaction terms. The forward stepwise procedure only kept two significant variables: foraging (1 degree of freedom khi2 = 7.449, p = 0,006) and treatment (1 degree of freedom khi2: nothing vs. 15 s, khi2 = 5.343, p = 0.021; but nothing vs. 30 s, khi2 = 0.056, p = 0.813). The odor factor and the two- and three-way interactions among the main factors were dropped during the stepwise procedure. This analysis indicates first that the two foraging groups (nectar and pollen) are different; in fact, as previously reported [17], [55], [90], [95]–[97] the pollen foragers overall performed better than the nectar foragers. Second, the 15 s group had a lower performance than groups that were either not treated or treated at 30 s. This corresponds to the group in which olfactory interference can be seen, so this is consistent with the analysis performed in Figure 4B. It also justifies our grouping of the “nothing” and “30 s” groups in Figure 4, as these groups are not different. (0.64 MB TIF) [file pone.0003513.s004.tif]

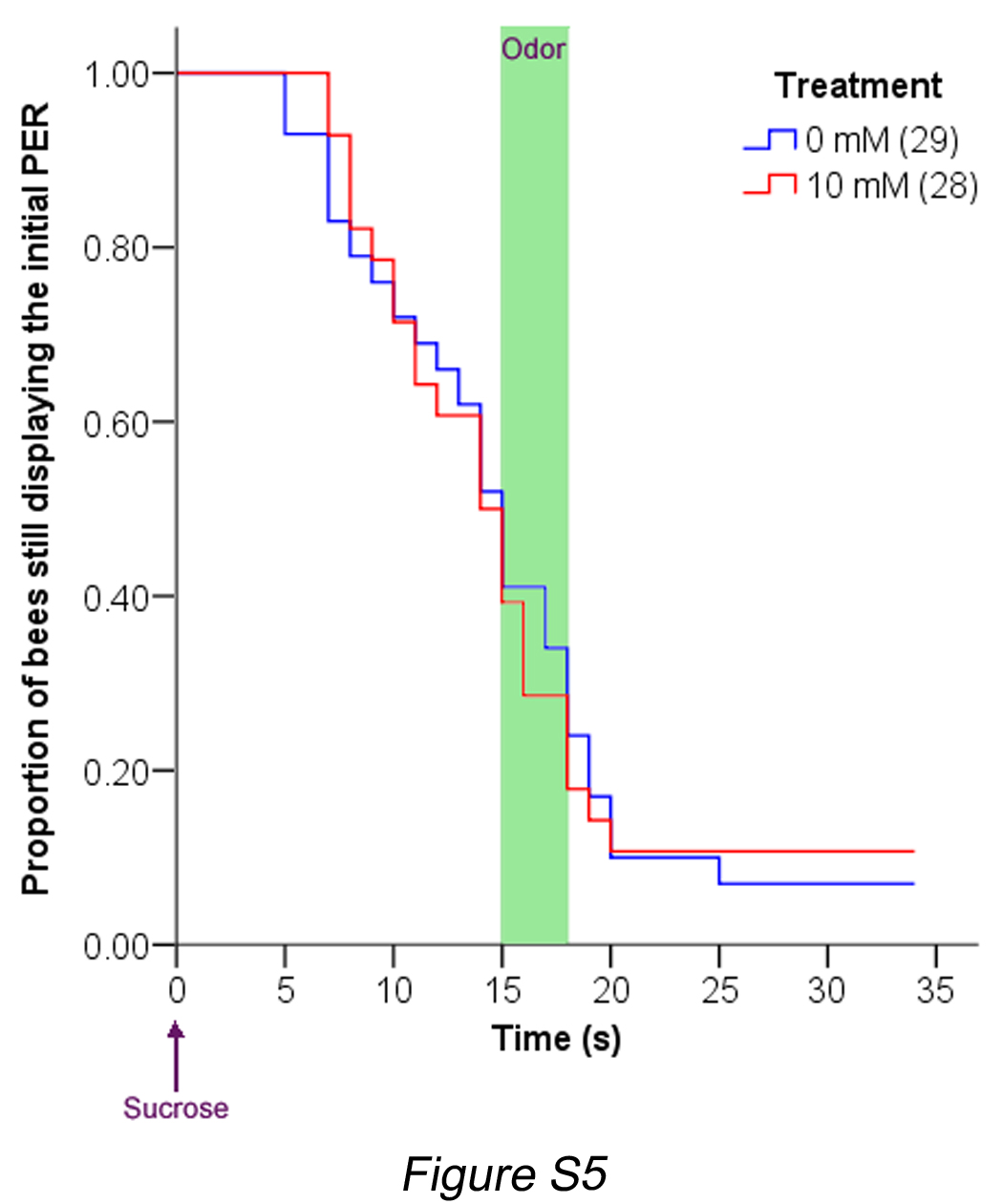

Supplement: Figure S5 — Effect of lobula injection of cimetidine on olfactory interference. To control the spatial specificity of the injection of cimetidine (Figure 6– 8), the olfactory interference experiment was replicated except that injection of the drugs was into the lobula of the optic lobe instead of into the deutocerebrum. The lobula is not involved in chemosensory processing or olfactory learning and is a standard control for the spatial specificity of treatments in the bee learning [62], [63], [70]. We used cimetidine (10 mM) and saline (0 mM, control group), and the odor was 1-nonanol. All other details are as in Figure 7A. Contrary to what was seen in Figure 7, animals injected with cimetidine in the lobula were not different from animals injected with saline solution (Wald test; time-period 0–14 s: khi2 = 0.001, p = 0.971; time-period 15–19 s: khi2 = 0.183, p = 0.669; time-period 20–34 s: khi2 = 0.652, p = 0.419). This confirms that cimetidine does not diffuse beyond its target, although it may affect both antennal lobe and dorsal lobe when injected into the deutocerebrum. (0.20 MB TIF) [file pone.0003513.s005.tif]

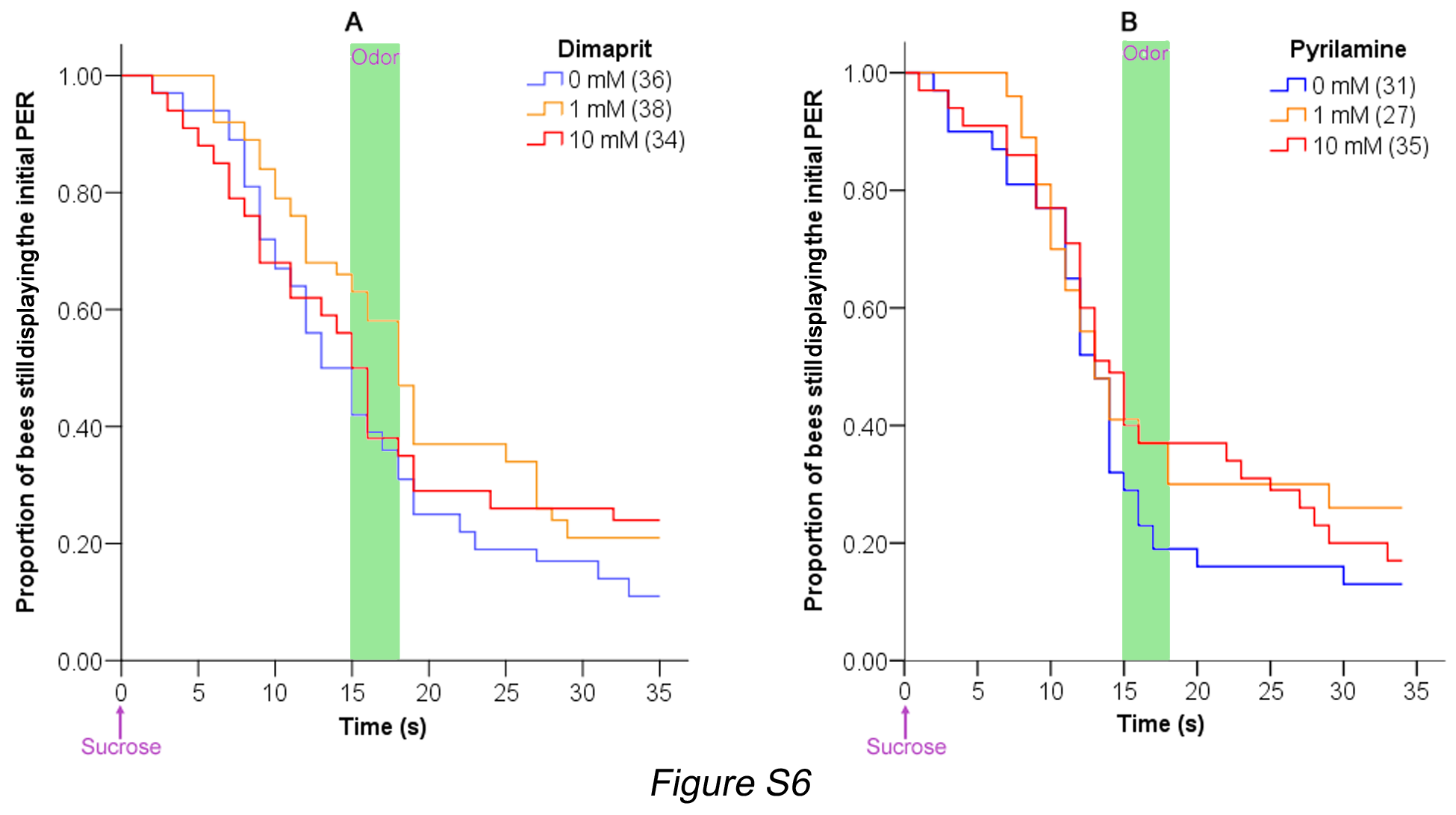

Supplement: Figure S6 — Effect of dimaprit (A) and pyrilamine (B) on olfactory interference. To further investigate the effect of histaminergic drugs, two other histamine receptor antagonists (dimaprit and pyrilamine) were injected to honey bees following the same protocol. Other details are as in Figure 7A. (A) Animals that received 10 mM or 1 mM dimaprit were not significantly different from the corresponding saline group (Wald test, p>0.050 in all cases). This suggests that dimaprit is less efficient than cimetidine at impairing olfactory interference. However, olfactory interference is not very clear in this case. (B) For pyrilamine, after the odor was presented, the 10 mM and the 1 mM groups maintained the PER for a longer time than the control group, and showed a trend for reduced olfactory interference. This is similar to the effect of cimetidine. However, neither of the pyrilamine groups was significantly different from the control group (Wald test, p>0.050 in all cases; in particular, for 0 mM vs. 10 mM in the 15–19 s time period, khi2 = 0.542, p = 0.461). In the fruit fly, pyrilamine is more efficient than dimaprit in blocking histaminergic receptors made of HisCl2 subunits (IC50 of 165 and 279 µM respectively, cimetidine being at 117 µM) while the opposite effectiveness is seen for histaminergic receptors made of HisCl1 subunits (IC50 of 442 and 56 µM respectively, cimetidine being at 21 µM; all these data from [85]). Therefore, our results suggests that the receptors involved in impairing olfactory interference involve the AmelHisCl2 subunit, which is the ortholog of fruit fly dimaprit-insensitive HisCl2 subunit [87]. (0.46 MB TIF) [file pone.0003513.s006.tif]
